# Standardized maps – an emerging approach to leverage quantitative information in knee imaging

**DOI:** 10.1016/j.ostima.2024.100251

**Published:** 2024-10-05

**Authors:** Paul Margain, Julien Favre, Brigitte M. Jolles, Patrick Omoumi

**Affiliations:** a Department of Musculoskeletal Medicine, Lausanne University Hospital and University of Lausanne (CHUV-UNIL), Lausanne, Switzerland; b The Sense Innovation and Research Center, Lausanne and Sion, Switzerland; c Institute of Microengineering, Ecole Polytechnique Fédérale de Lausanne (EPFL), Lausanne, Switzerland; d Department of Diagnostic and Interventional Radiology, Lausanne University Hospital and University of Lausanne (CHUV-UNIL), Lausanne, Switzerland

**Keywords:** Maps, Knee, Imaging, Osteoarthritis, Cartilage thickness, Cartilage composition, Bone mineral density, Bone shape, Segmentation, Registration, Standardization, atlas

## Abstract

**Objective:**

Property maps, which capture spatial variations across the entire joint, are emerging as a powerful means for extracting and analyzing quantitative information from knee 3D imaging datasets, particularly from CT and MRI data. This perspective paper aims to discuss the processing pipelines used so far, as well as the results they have enabled with respect to osteoarthritis.

**Design:**

The key methodological steps for obtaining property maps, including segmentation, property calculation, and standardization are presented and analysis methods are discussed. Representative studies are also examined to illustrate the state-of-the-art in this field.

**Results:**

Three main processing pipelines have been used, with the segmentation, property calculation, and standardization steps occurring in different orders. Many methods have been successfully considered for ordering these steps, without any looking generally preferable to the others. Thanks to recent advances in segmentation and standardization techniques, routine processing of property maps appears conceivable in the near future. Maps have been analyzed for multiple purposes, including group comparisons, pattern recognition, and cross-property modelling. Mostly maps of cartilage thickness and composition, as well as maps of bone shape and mineral density have been reported. They revealed distinct patterns associated with osteoarthritis severity, achieved high diagnostic accuracy, and identified relationships among tissue properties.

**Conclusions:**

Property maps represent a promising approach for leveraging the extensive information in imaging data. They are particularly interesting for standardizing complex spatial variations in tissue properties, enabling global analysis and modelling. Once challenging to obtain and interpret, current mapping methods are being improved to the point that property maps may well be in routine use in the near future.

Osteoarthritis (OA) of the knee is a complex disease affecting the whole joint [1]. Imaging has allowed the identification and characterization of multiple structural alterations, including in cartilage morphology and composition, as well as in bone shape and mineral density [2]. Along with semi-quantitative assessment approaches, these properties have been commonly quantified using their average value in regions of interest. In CT imaging, for example, this might involve measuring Hounsfield units in a specific bone region, or in MRI, it could include regional measures of cartilage thickness or compositional parameters, like T2 relaxation times. While this approach provides important quantitative data, it tends to neglect the spatial variations of the properties across the entire joint. To analyze these spatial variations and to avoid the limitations of regional analysis of properties that are otherwise continuous, some authors started using property maps, as illustrated in Fig. 1. Such maps, once challenging to obtain and interpret, are now more readily accessible thanks to improvements in image segmentation, standardization, and map analysis methods. These advances set the basis for the routine use of property maps in the future. Given this background, this work reviews the solutions presented in the literature regarding the key methodological steps leading to the creation of property maps. To illustrate the manuscript, six representative studies of the state-of-the-art were selected and discussed throughout the review [[3], [4], [5], [6], [7], [8]] (Table 1).

**Fig. 1:**
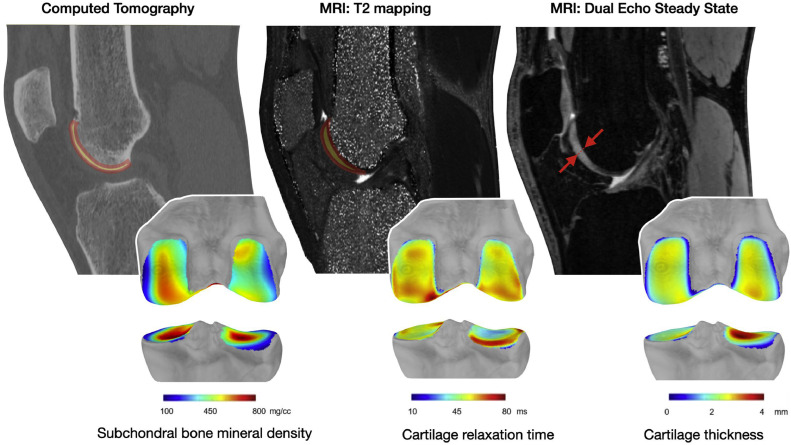
Three examples of property maps from computed tomography (CT) and magnetic resonance imaging (MRI) 3D scans. In addition to illustrating the resolution current methods can achieve, these maps highlight the unneglectable spatial variations (property distribution across the entire tissue surface) that exist in subchondral bone mineral density, cartilage relaxation time, and cartilage thickness. The red arrows indicate the thickness of the cartilage. Data from the Lausanne Knee Study (LKS).

**Table 1:** Overview of the methods in representative studies of the state-of-the-art use of maps to analyze tissue properties in knee OA.

Reference	Property of interest	Dataset	Segmentation	Property calculation	Standardization	Map analysis
[3]	Cartilage thickness	*N* = 175 MRI: SPGR MRI	Manual	Surface-to-surface distance	Registration to a 3D atlas; 2D projection	Statistical Parametric Mapping
[4]	Cartilage thickness	*N* = 6265 MRI: DESS*	Automatic (3D U-Net)	Surface-to-surface distance	Image registration; 2D projection	Angle based Joint and Individual Variation Explained (AJIVE) analysis
[5]	Cartilage thickness, and subchondral bone mineral density	*N* = 50 CT arthrogram	Manual	Surface-to-surface distance and average of CT voxels values	Registration to a 3D atlas; 2D projection	Maximum localization
[6]	Bones shape	*N* = 41,822 MRI: DESS *	Automatic (3D V-Net)	Distance to common referential origin	Spherical 2D transformation	Convolutional neural network model (ResNet)
[7]	Bones shape, cartilage composition, and thickness	*N* = 7437 MRI: DESS & MSME*	Automatic (3D V-Net)	Distance to common referential origin, average of T2 voxel values and Euclidean distance transform along the morphological skeleton of the masks	Spherical 2D transformation	Convolutional neural network model (ResNet), and Class Activation Maps (Grad-CAM)
[8]	Cartilage composition	*N* = 4384 MRI: MSME MRI*	Registration to already segmented images	Sampling of T2 voxel values	2D flattening technique	Convolutional neural network model (DenseNet)

⁎ Osteoarthritis Initiative (OAI) dataset.

## Processing pipelines

**Fig. 2:**
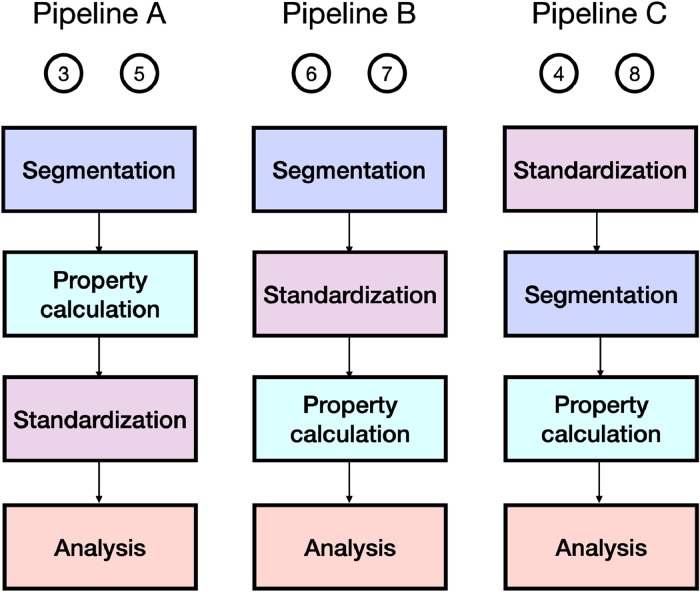
Illustration of the three processing pipelines (A-C) commonly used to obtain and analyze property maps, and which are examined in this study. The circled numbers at the top of the pipelines refer to published studies listed in the references section.

The methods leading to property maps in the literature share some processing steps, such as segmentation, property calculation and standardization. Their order, however, varies among the studies. Most reporting so far has focused on just three processing pipelines, which we have labeled A, B, and C (see Fig. 2).

## Segmentation

A key step of all processing pipelines is the segmentation of the tissues of interest in the imaging data. Currently, high-resolution 3D datasets are best suited for property mapping due to their superior spatial resolution, which allows more detailed capture of the spatial variations across the joint.

With pipelines A and B, segmentation occurs first. The gold standard technique in this case remains manual or semi-manual delineation of the tissues by expert operators, often radiologists [3,5,9]. This time-consuming approach may be appropriate for small studies, but would be unworkable when there are large volumes of data

The recent advent of artificial intelligence in medical imaging has significantly transformed the tools available for segmentation. Convolutional neural networks and especially the U-Net architecture, which is a deep learning model tailor-made for segmentation challenges [10], have reduced the reliance on expert manual processing and enabled more scalable analysis. Already about half of the publications on property maps have used these novel methods to segment the bone and cartilage tissues of the knee [4,6,7]. Although they decrease the reliance on an operator and allow the analysis of large datasets, these novel methods still rely on supervised training with a subset of manually segmented data. By analogy to other fields, for example with natural images [11], we can anticipate the development of comprehensive foundational models for medical image segmentation in the near future. We can also expect more advanced approaches, such as transformers or diffusion models [11]. These advances are expected to boost the use of property maps, as one of the most important barriers to their wide adoption is the extensive segmentation effort they require using current tools. It is important to note that while artificial intelligence-based segmentation tools offer great promise, they will still require thorough validation before they can be relied on in research or clinical settings [12].

With pipeline C, segmentation occurs after standardization. Since standardization, in this case, consists of registering the images from a knee of interest to reference images already (manually) segmented [8], the segmentation becomes a formality. Indeed, segmentation of the reference images can be directly used for the registered images.

## Property map calculation

Calculating the maps is rather straightforward, and the different methods are generally independent of the processing pipeline. Furthermore, the methods used so far are mostly extended versions of those previously proposed to calculate tissue properties in studies based on regions of interest. Usually, the maps are obtained in two phases. First, the segmentations (standardized (B and C) or not (A)) are combined to reconstruct 3D models of the tissues, using methods such as marching cubes [4,7] or 3D point cloud conversion [6].The properties are then calculated for each point of the 3D bone models. Cartilage thickness was determined by calculating the distance from the bone surface to the cartilage surface [[3], [4], [5]] or with the projection of a morphological skeleton [7]. For cartilage composition and bone mineral density, the values of the voxels in the vicinity of the bone were averaged over small volumes [5,7]. These calculations resulted in bone models augmented with the values of the properties of interest (Fig. 1). These augmented models form the property maps. If a standardization procedure occurs before the calculation of the property maps, as in B and C, standardized maps are obtained directly. If the processing pipeline does not include a standardization procedure beforehand, as with A, the maps are subject-specific, meaning that they are based on bone models that differ among individuals and possibly among time points. To enable cross-individual or longitudinal analyses, the maps need to be standardized afterward.

## Standardization

Two standardization approaches were considered depending on which processing pipeline was being used. With pipelines A and B, the standardization is based on the registration of the 3D model or 3D map against an atlas [3,[5], [6], [7]]. Various algorithms have been used to calculate the registration transformation, including Iterative Closest Point (ICP) [6] and Thin Plates Splines (TPS) [3,5]. Once the transformation has been determined, the property values of the map are transferred to the atlas model (A) or calculated directly using the atlas (B). For C, standardization consists of registering the images of the knee to reference images based on global image similarity, and then using these standardized images to calculate the maps [4,8]. Usually, when the method results in a 3D standardized map, a 2D transformation is applied to create a standardized 2D property map [[3], [4], [5]].

Standardization based on registration against a 3D atlas uses information from the tissue of interest exclusively. This focused approach may better preserve spatial correspondence between tissues in different spaces. There is certainly, however, no method of standardization, or order of processing that is always better than the others, as the effectiveness of the method is context dependent.

## Property map analysis

Once standardized and expressed in 2D, the maps are mathematically equivalent to images. Consequently, a range of analysis methods can be applied independently of the processing pipelines. To better present these possibilities, from comparison and pattern recognition to cross-properties analysis, this section reviews the methods as well as the results so far.

Regarding comparison analysis, for example, one study [3] compared cartilage thickness maps at various stages of medial-compartment OA using statistical parametric mapping. This study revealed distinct thickness patterns for each clinical stage, forming a coherent progression from the non-OA to the severe OA subgroups. As disease severity increased, patterns emerged, notably thinner cartilage in the anterior area of the medial femoral condyle and tibia compartments. More recently, another study using a different processing pipeline, but the same comparison method, observed similar consistent patterns [4].

The extensive information provided by the maps allows detailed analysis of spatial variations in knee properties, facilitating pattern recognition analysis. For instance, convolutional neural networks have been trained to recognize these patterns in the maps for OA prediction and diagnosis. One such study applied a classification model (ResNet) to bone shape maps and achieved a notable area-under-the-curve of 0.905 for OA diagnosis [6]. Another example comes from a study that analyzed the composition of the cartilage matrix using maps of T2 relaxation times [8].The study compared the capacity of demographic and relaxation time maps to distinguish between knees with or without radiographic OA. It reported that a convolutional neural network (DenseNet) trained on relaxation time maps significantly outperformed a common classifier using demographic data. These two examples in particular demonstrate well the relevance of spatial variations in tissue properties as captured and expressed by the standardized maps.

Because osteoarthritis involves interactions among many tissues, studying the relationships among knee properties has drawn much interest. Maps have also proven to be a valuable tool for cross-property analysis. In maps of non-OA femoral cartilage thickness and bone mineral density, for example, a relationship was found between the locations of the thickest cartilage and the locations of the densest bone, supporting the concept of a functional cartilage/subchondral bone unit [5].

Finally, maps may help to identify areas of particular interest. This is well illustrated by a study that used deep learning models (Grad-CAM) to correlate pain with local features of cartilage thickness, bone shape, and cartilage composition maps [7].

## Discussion and outlook

Looking to the future, property maps hold potential for both research and patient care in the context of OA. For example, they could be used in clinical trials to track knee property changes. Their standardized nature allows for consistent, quantitative comparisons over time and among cohorts, potentially increasing the sensitivity and reliability of trial outcomes. Extending from recent work, we could also imagine the maps used as preprocessed data for property scores. Property maps might also play an important role in personalized medicine. By providing a more interpretable and quantifiable representation of joint health compared to raw images, these maps could aid clinicians in early disease detection, risk assessment, and treatment planning. Subtle changes in cartilage thickness or bone mineral density patterns, for example, might be more readily apparent and quantifiable in map form, allowing more individualized management. Furthermore, as machine learning and artificial intelligence continue to advance, standardized maps could serve as ideal inputs for developing predictive models. For example, we could envisage models to help identify patients at higher risk of rapid disease progression or to predict individual responses to specific treatments, thereby enabling more focused and effective interventions.

Property maps bridge a gap between complex imaging data and clinically actionable information. These days, a milestone has been passed, as methods exist to integrate them into both research and clinical workflows. We can be confident that once available in large datasets, property maps will prove useful in various contexts suggested above.

In conclusion, the journey toward understanding OA has benefited significantly from the evolution of imaging technologies in recent decades and the emergence of property maps suggests that this evolution will continue. Advances in processing methods, particularly in segmentation, standardization, and map analysis, have transformed the maps from a dream only 20 years ago [13] to reality today. Property maps promise to leverage the extensive information in imaging data. They may prove especially relevant for extracting the important information contained in spatial variations. Different analytical strategies have been developed, generating new information through pattern recognition, cross-sectional or longitudinal comparisons, or cross-property modelling. With these openings, quantitative imaging can further contribute to the understanding of OA pathophysiology, and eventually help to enable early disease detection and more effective prevention strategies.

## CRediT authorship contribution statement

**Paul Margain:** Writing – original draft, Visualization, Writing – review & editing. **Julien Favre:** Writing – original draft, Visualization, Writing – review & editing. **Brigitte M. Jolles:** Writing – review & editing. **Patrick Omoumi:** Writing – review & editing.

## Declaration of competing interest

None.
